# Budesonide/glycopyrronium/formoterol fumarate triple therapy prevents pulmonary hypertension in a COPD mouse model via NFκB inactivation

**DOI:** 10.1186/s12931-022-02081-y

**Published:** 2022-06-27

**Authors:** Ratoe Suraya, Tatsuya Nagano, Gusty Rizky Teguh Ryanto, Wiwin Is Effendi, Daisuke Hazama, Naoko Katsurada, Masatsugu Yamamoto, Motoko Tachihara, Noriaki Emoto, Yoshihiro Nishimura, Kazuyuki Kobayashi

**Affiliations:** 1 Division of Respiratory Medicine, Department of Internal Medicine, Kobe University Graduate School of Medicine, 7-5-1 Kusunoki, Chuo, Kobe, Japan; 2 Laboratory of Clinical Pharmaceutical Science, Kobe Pharmaceutical University, 4-19-1 Motoyama Kitamachi, Higashinada, Kobe, Japan; 3 Division of Cardiovascular Medicine, Department of Internal Medicine,, Kobe University Graduate School of Medicine, 7-5-1 Kusunoki, Chuo, Kobe, Japan

**Keywords:** Chronic obstructive pulmonary disease, Pulmonary hypertension, Budesionde, Glycopyrronium, Formoterol fumarate, NFκB

## Abstract

**Background:**

Chronic obstructive pulmonary disease (COPD) is a health problem that results in death, commonly due to the development of pulmonary hypertension (PH). Here, by utilizing a mouse model of intratracheal elastase-induced emphysema that presents three different phases of COPD, we sought to observe whether budesonide/glycopyrronium/formoterol fumarate (BGF) triple therapy could prevent COPD-PH in addition to ameliorating COPD progression.

**Methods:**

We utilized intratracheal elastase-induced emphysema mouse model and performed experiments in three phases illustrating COPD progression: inflammatory (1 day post-elastase), emphysema (3 weeks post-elastase) and PH (4 weeks post-elastase), while treatments of BGF and controls (vehicle, one-drug, and two-drug combinations) were started in prior to elastase instillation (inflammatory phase), at day 7 (emphysema), or at day 14 (PH phase). Phenotype analyses were performed in each phase. In vitro, A549 cells or isolated mouse lung endothelial cells (MLEC) were treated with TNFα with/without BGF treatment to analyze NFκB signaling and cytokine expression changes.

**Results:**

We observed significant reductions in the proinflammatory phenotype observed in the lungs and bronchoalveolar lavage fluid (BALF) 1 day after elastase administration in mice treated with BGF compared with that in mice administered elastase alone (BALF neutrophil percentage, p = 0.0011 for PBS/Vehicle vs. PBS/Elastase, p = 0.0161 for PBS/Elastase vs. BGF). In contrast, only BGF treatment significantly ameliorated the elastase-induced emphysematous lung structure and desaturation after three weeks of elastase instillation (mean linear intercept, p = 0.0156 for PBS/Vehicle vs. PBS/Elastase, p = 0.0274 for PBS/Elastase vs. BGF). Furthermore, BGF treatment prevented COPD-PH development, as shown by improvements in the hemodynamic and histological phenotypes four weeks after elastase treatment (right ventricular systolic pressure, p = 0.0062 for PBS/Vehicle vs. PBS/Elastase, p = 0.027 for PBS/Elastase vs. BGF). Molecularly, BGF acts by inhibiting NFκB-p65 phosphorylation and subsequently decreasing the mRNA expression of proinflammatory cytokines in both alveolar epithelial and pulmonary endothelial cells.

**Conclusion:**

Our results collectively showed that BGF treatment could prevent PH in addition to ameliorating COPD progression via the inhibition of inflammatory NFκB signaling.

**Supplementary Information:**

The online version contains supplementary material available at 10.1186/s12931-022-02081-y.

## Background

Chronic obstructive pulmonary disease (COPD) has been a major health problem, especially in the past decade, with an estimated mean prevalence of 13.1% in all populations worldwide [1, 2]. In addition, COPD has also been considered the third-highest cause of death, and a reported 3.23 million deaths have been attributed to COPD [3]. One of the main complications that contributes to COPD-related death is the eventual development of pulmonary hypertension (PH); PH develops in approximately 8% of all COPD patients [4]. COPD-PH is categorized in Group 3 of the WHO classification of PH, which includes PH caused by hypoxia or lung diseases [5]. Aberrant pulmonary vasoconstriction and subsequent vascular remodeling through the neomuscularization of precapillary arteries are integral causes of PH development [6–8]. Hypoxia-hypoxemia due to obstructed airflow is known to induce vascular remodeling in COPD-PH, as is known for other conditions that cause Group 3 PH; however, unlike other classes of PH, such as PAH, for which PAH-specific drugs are available, few, if any, drugs have been shown to successfully target and treat COPD-PH [9, 10]. As such, there is an urgent need to develop novel treatment options for this specific condition.

Recently, a novel inhalation drug that combines three different drug classes (inhaled corticosteroids/ICS, long-acting beta agonists/LABA, and long-acting muscarinic antagonists/LAMA) was shown to improve lung function in COPD patients [11]. One of these triple inhalation therapies includes budesonide (B), glycopyrronium (G), and formoterol (F), which are the most widely used inhalation drugs and have already been established as staples of COPD therapy. The clinical effects of this triple therapy have also been well established [11]. Furthermore, the individual classes of drugs that constitute BGF therapy have been associated with positive effects in pulmonary vasculature cells, in which they have been reported to affect molecules (e.g., nitric oxide (NO) or vascular endothelial growth factor (VEGF)) and processes (e.g., vasodilation, angiogenesis, and vascular cell proliferation) related to pulmonary vascular tone and vascular remodeling, suggesting their potential benefit in treating PH, specifically COPD-PH [12–15].

Of note, a prominent and relatively established pathway that controls vascular tone and the subsequent remodeling process is the cytokine-mediated inflammatory pathway [6, 8, 16, 17]. Vascular inflammation has been shown to contribute to the dysfunction of pulmonary vascular cells, such as endothelial cells (ECs) and smooth muscle cells [14, 15, 18]. It has been reported that NFκB signaling plays a significant role in the development of pulmonary hypertension; activation of this particular pathway is widely observed in many types of pulmonary vascular cells, and its inhibition can ameliorate the PH phenotype [19–21]. In addition, the expression of various proinflammatory cytokines, including but not limited to TNFα, IL-6, IL-1β, CCL1, CCL2, and CCL8, is upregulated in PH conditions [15, 17, 19, 21–23]. Moreover, similar inflammatory signaling molecules and pathways play a significant and established role in the pathogenesis of COPD, thus revealing a common, underlying pathological link between both COPD and PH [3, 4, 6, 7, 16]. As such, targeting this inflammatory pathway could become a viable strategy for treating both conditions efficiently.

The use of triple inhaled therapies, such as BGF, or even other possible combinations of ICS/LAMA/LABA drug classes in treating COPD-PH, as well as Group 3 PH, has not been established, and the molecular mechanism underlying the effects of these combination drugs are not known. The aim of this study was to elucidate whether these drugs, especially when combined, could help treat PH in addition to COPD. As such, in this study, we hypothesized that BGF combination therapy could act to prevent the development of pulmonary hypertension in COPD, and we sought to prove this hypothesis accordingly.

To this end, an in vivo mouse model of emphysema established via intratracheal elastase instillation that was terminated at different time points was used to better illustrate the pathological progression toward COPD-PH. We first showed that BGF treatment is effective in treating elastase-induced emphysema both in the acute inflammatory phase 1 day after elastase treatment and in the emphysematous phase 3 weeks after elastase treatment. We further proved that BGF treatment is also beneficial in preventing PH development in elastase-treated mice, as shown by improved hemodynamics, right ventricular hypertrophy, and histological pulmonary vascular remodeling. Additionally, in vitro, we analyzed the role of NFκB transcription after BGF treatment.

## Methods

### Animal studies and in vivo experimental protocol

BALB/c mice were purchased from CLEA Japan and maintained in-house. All the animals were provided food and water ad libitum and were maintained under a 12-h dark–light cycle. For all the experiments, we used 8- to 10-week-old female mice. To establish emphysema in mice, we administered intratracheal instillations of elastase (WAKO Fujifilm, Japan) (3 U/mouse) or a PBS negative control to BALB/c mice, which will be subject to further phase-specific treatment protocol. Elastase instillation was performed under intraperitoneal anesthesia. For the elastase-treated groups, the mice were further divided into groups to receive various treatment combinations [the B (500 ng/mouse), G (15.36 ng/mouse), F (23.04 ng/mouse), BF, GF, and BGF groups] or vehicle as a control. These drugs were administered intraperitoneally. The timing of treatment administration was adjusted in accordance with the experimental phase (15 min prior to elastase instillation for the inflammatory phase, 3 × weekly beginning on Day 7 for the emphysematous phase, and 3 × weekly beginning on Day 14 for the PH phase). The mice were terminated 24 h after elastase administration for inflammatory phase, at Day 14 for the emphysematous phase, and at Day 28 for the PH phase. For tissues subject to further histological analysis, after termination and perfusion with PBS, mouse lungs were fixed with 10% formalin for 24–48 h before being processed into paraffin blocks and subsequently into histological sections [24].

### Bronchoalveolar lavage fluid collection and cytology analysis

After termination of the mice, bronchoalveolar lavage (BAL) fluid was collected as previously described [25]. Briefly, after termination, 800 μL PBS was inserted and reaspirated via trachea three times using a blunted 18G needle and centrifuged at 1500 rpm for 10 min. Then, 1500 μL was removed and stored, the sample was mixed again, and 50 μL was processed using Cytospin as per manufacturer’s protocol. Cytology analysis images of BAL fluid smears were captured by a Keyence BZ-X800 microscope (Keyence), and the cells were counted up to 200 before proceeding to differential blood count analysis. For the total cell count, before the BAL fluid was centrifuged, 10 μL of the fluid was collected and mixed with 10 μL Turk’s solution and inserted into a hemocytometer before manually counting the cells.

### Peripheral oxygen saturation examination

According to the manufacturer's recommendation, the oxygen saturation level during the emphysema phase was measured via a paw sensor on Days 0 (prior to elastase instillation), 2, 9, and 16 using MouseSTAT Jr (Kent Scientific, Torrington, CT, USA) on conscious mice. Five 10-s readings were averaged and presented as the results.

### Histological staining and MLI analysis

The sections were stained with hematoxylin–eosin staining as previously described [25]. For mean linear intercept (MLI) analysis, measurements were taken from three randomly selected fields of view/sample (20 × magnification) and averaged the results. We performed the measurement in three biologically independent samples per group using semiautomated macros in ImageJ software as previously described [26].

### Echocardiography

We performed mouse transthoracic echocardiography using a Siemens Acuson X300 connected to a VF13-5SP probe (Siemens) as previously described. Pulmonary artery acceleration time (PAAT) was measured three times and averaged [27].

### Right heart catheterization

Right heart catheterization was performed as previously described [27]. Right heart catheterization analyzing right ventricular systolic pressure (RVSP) was performed by inserting a 1.4 F Millar catheter into the right jugular vein under anesthesia with 2% isoflurane immediately before the mice were terminated. The RVSP was calculated from the average of five pressure waves.

### Fulton index measurement

Fulton index measurements were performed as previously described [27]. Fulton index measurement on mouse hearts after termination by measuring the weight of the right ventricle and normalizing this value to the weight of the left ventricle and septum. Data were presented as the right ventricle/left ventricle + septum weight.

### Immunostaining

Immunostaining of α-smooth muscle actin (α-SMA) and von Willebrand Factor (vWF) was performed and analyzed as previously described [27–29]. Anti-von Willebrand factor (1:250, Abcam), FITC-labeled anti-α-smooth muscle (1:250, Sigma–Aldrich), eNOS (1:250, abcam), VEGF-A (1:100, abcam), and Ki67 (1:200, abcam) antibodies were used as the primary antibodies. Images were captured with a Keyence BZ-X800 fluorescence microscope. A precapillary pulmonary artery (20–50 μm in diameter) was considered to be partially muscularized when 25–75% of the total diameter of the artery (determined by the vWF-positive cells) was α-SMA-positive, and it was considered fully muscularized when 75–100% of the total artery diameter was α-SMA-positive. Pulmonary arteries from 4 random images (20 × magnification) of each sample were counted, and these numbers were normalized to the total number of arteries in each picture and averaged. Immunostaining of eNOS, VEGF-A, and Ki67 were performed as previously described. Briefly, after deparaffinization and antigen retrieval, sections were blocked in 3% H_2_O_2_, Avidin and Biotin blocking reagent (Vector), 4% Donkey Serum in PBS, alternating with PBS washes. First antibody incubation was performed overnight at 4 °C. The next day, blocking with ABC kit (Vector) prior to color development using ImmPACT DAB substrate (Vector) were performed until optimal color was achieved. Slides were then stained with hematoxylin, mounted, and analyzed.

### Mouse lung endothelial cell (MLEC) isolation and cell culture

MLEC isolation was performed using the magnetic sorting system as previously described with slight modification [27, 30]. Briefly, lung specimens were harvested and minced manually using scissors prior to digestion using a mouse lung dissociation kit (Miltenyi Biotec). Then, the samples were sorted with anti-CD146 magnetic microbeads (Miltenyi Biotec) as previously reported. The resulting isolated EC suspension was then plated and cultured in fully supplemented HuMedia-EG2 (Kurabo) supplemented with 1% penicillin/streptomycin until the cells reached ~ 90% confluence. Then, the cells were treated with TNFα (10 ng/mL) and/or a combination of B (10 nM/mL), G (10 nM/mL) and F (10 nM/mL). In addition, A549 cells were cultured in RPMI-1640 (Gibco) supplemented with 10% FBS (Gibco) and 1% penicillin/streptomycin (Gibco) until they reached ~ 90% confluence; then, the cells were treated with TNFα/BGF.

### Real-time quantitative PCR

Lung samples were homogenized in RNAlater (Qiagen), while cell samples were harvested in Sepasol RNA I Super G (Nacalai Tesque). Further RNA isolation, cDNA was synthesized using the PrimeScript One-Step RT–PCR kit (Takara), and real-time qPCR was performed as previously described [25]. The primers used are listed in Additional file 1: Table S1.

### Immunoblotting

Immunoblotting was performed as previously described [25]. For each sample, 20 μg of protein/sample were inserted into each well. The antibodies used were anti-NF-kB-p65 (CST; 1:1000), anti-phospho-NF-kB-p65 (CST; 1:500), anti-GAPDH (CST; 1:1000) and anti-β-actin (CST; 1:1000) as the primary antibodies and anti-rabbit HRP-linked IgG antibodies (CST; 1:3000) as the secondary antibodies. The antibodies were diluted in Can Get Signal immunoreaction enhancer solution (Toyobo) and detected with Amersham ECL select (Cytiva).

### Statistical analysis

The data are presented as the mean ± standard error of the mean (SEM). Differences between two groups were analyzed using a two-tailed Student’s t test, and differences among three or more groups were analyzed with a one-way ANOVA test with Tukey’s post-hoc test. *P* values of < 0.05 were considered significant. All the statistical analyses were performed using GraphPad Prism 8.0.1 (GraphPad Software Inc.).

## Results

### BGF and GF ameliorate elastase-induced airway and lung inflammation

**Fig. 1:**
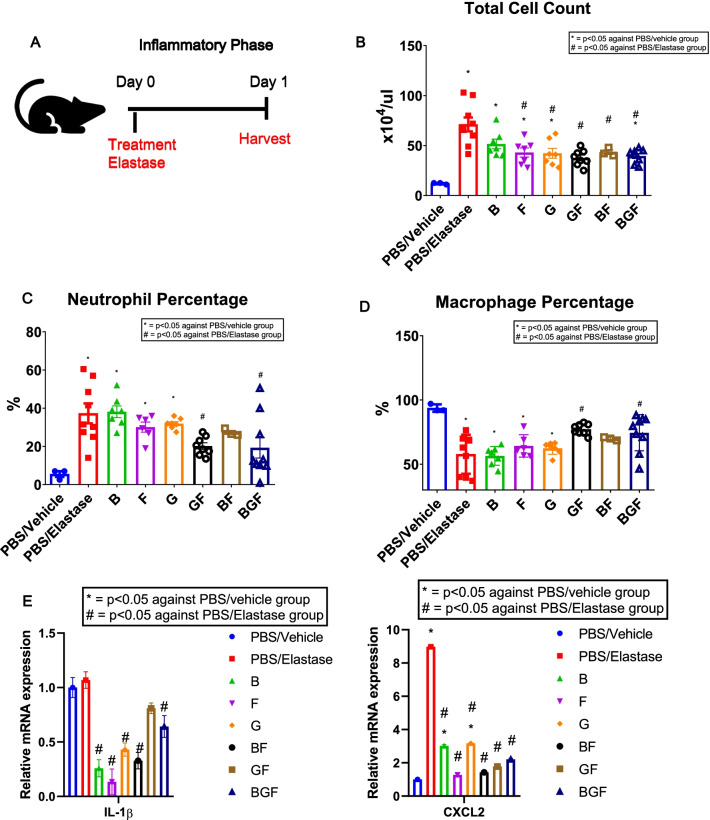
BGF and GF treatments protect against lung inflammation 24 h after elastase instillation. **A** Schematic diagram of the experimental setup for the inflammatory phase. BALB/c mice were treated with drug combinations or vehicle as a control 15 min prior to intratracheal elastase instillation and sacrificed after 24 h. Bronchoalveolar lavage (BAL) fluid or lung tissue was collected at termination. **B**–**D** Cytological analysis of BAL fluid from all the experimental groups, including total cell count (**B**), neutrophil percentage (**C**) and macrophage percentage (**D**). **E** Relative mRNA expression levels of IL-1β and CXCL2 in lung tissue 24 h after elastase or PBS instillation. n = 3–9 mice per group for (**B**–**D**) and n = 3 per group for (**E**). The data are presented as the mean ± S.E.M. (standard error of the mean). One-way ANOVA with Tukey’s post-hoc test was used for all figures. *, p < 0.05 compared with the PBS/Vehicle group, #, p < 0.05 compared with the PBS/Elastase group. B, budesonide; G, glycopyrronium; F, formoterol; BF, budesonide and formoterol; GF, glycopyrronium and formoterol; BGF, budesonide, glycopyrronium and formoterol

We first sought to confirm the efficacy of BGF treatment in ameliorating the acute inflammatory phase commonly seen in COPD by administering intratracheal instillation of elastase (3 U/mouse) or PBS as a negative control in BALB/c mice. Fifteen minutes prior to elastase instillation, we administered various treatment combinations (B, G, F, BF, GF and BGF) or vehicle as a control. After 24 h, the mice were sacrificed and harvested for phenotype analysis (Fig. 1A).

We first collected BALF from mice in all the experimental groups and performed cytological analysis of BALF smears to assess the acute inflammatory condition of the lung. Cytology analysis of BALF revealed an increase in the total cell counts after elastase treatment, while treatment with G, F, BF, GF and BGF significantly decreased the elastase-induced increase in cell counts (Fig. 1B). Furthermore, we analyzed the percentages of neutrophils and macrophages, and we found a significant increase in the neutrophil percentage (Fig. 1C), with a subsequent decrease in the macrophage percentage (Fig. 1D) and no significant difference in lymphocyte percentage (Additional file 2: Fig. S1) after elastase treatment compared to PBS/vehicle treatment. Compared with the other drug treatments, only the GF and BGF treatments significantly ameliorated the increase in the neutrophil percentage and the subsequent decrease in the macrophage percentage.

To strengthen our analysis of the inflammatory state, we performed real-time quantitative PCR analysis of the lung tissue and analyzed the mRNA expression levels of several proinflammatory cytokines. We found that the elastase-induced increase in the levels of IL-1β and CXCL2, which are cytokines related to COPD progression and inflammatory processes, were ameliorated in all of the treatment groups, including the BGF treatment group (Fig. 1E). These results collectively showed that the BGF and GF treatments could consistently reduce the proinflammatory state observed in the acute phase of COPD in vivo.

### Emphysematous lung condition after 3 weeks of elastase induction is reduced by BGF treatment

**Fig. 2:**
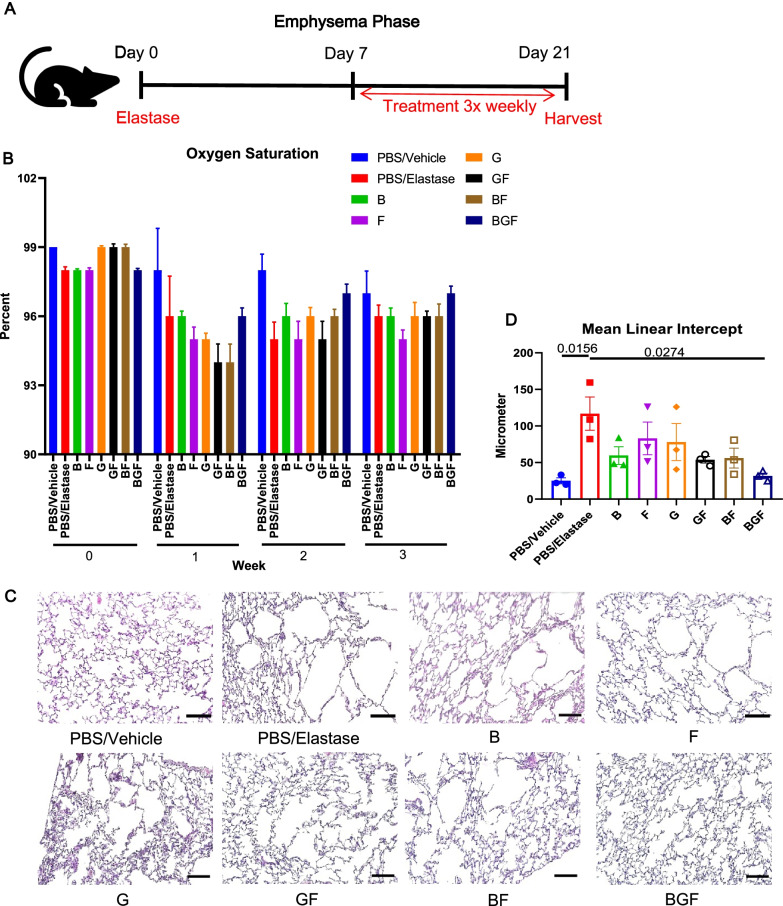
BGF treatment ameliorates the emphysematous lung phenotype after elastase instillation. **A** Schematic diagram of the experimental setup for the emphysematous phase. BALB/c mice were treated with drug combinations or vehicle on Day 7 after elastase instillation and sacrificed on Day 21. Lung tissues were fixed at termination for further analysis. **B** Peripheral oxygen saturation level of the mice (n = 3–8 mice) in each experimental group were assessed at each week after elastase instillation. **C** Representative hematoxylin–eosin staining of histological lung sections from each experimental group. Scale bar: 100 μm. **D** Quantification of the mean linear intercept (MLI) of hematoxylin–eosin-stained histological sections from all the experimental groups. n = 3 mice per group. The data are presented as the mean ± S.E.M. (standard error of mean). One-way ANOVA with Tukey’s post-hoc test was used for (**D**)

A later phase of COPD is the emphysematous phase, during which functional and structural changes in the lung alveolar bed cause air trapping, inhibit normal oxygen exchange, and cause a chronic hypoxemia-hypoxia state. To evaluate whether BGF treatment could positively affect this emphysematous condition, we administered similar single-dose intratracheal instillations of elastase or PBS to BALB/c mice and sacrificed the mice after 21 days (Fig. 2A). At Day 7 after elastase instillation, the mice were treated with drug combinations or vehicle, similar to treatments during the inflammatory phase, three times weekly until sacrifice (Fig. 2A).

To first assess whether there was any effect of treatment on the hypoxic-hypoxemic condition of the mice after elastase instillation, we analyzed the peripheral oxygen saturation levels of the mice at Days 0 (prior to elastase instillation as baseline), 2, 9, and 16 (Fig. 2B). On Day 2, the mice in most experimental groups showed a downward trend in oxygen levels that did not improve on Day 9, when drug administration had just been initiated. At Day 16, the PBS/elastase group did not show a complete recovery to a level similar to that in the PBS/vehicle-treated negative control group, the BGF/elastase group successfully reached an oxygen saturation level similar to that in the PBS/vehicle group (Fig. 2B).

Further analysis of histological lung sections revealed a highly disrupted alveolar structure three weeks post-elastase instillation, and this effect was most visible in the PBS/elastase group (Fig. 2C). Improvement of the lung structure could be seen in the double-drug and BGF-treated groups, with the BGF group showing marked preservation of the alveolar structure. To objectively analyze this result, we semiautomatically measured the mean linear intercept (MLI) of all the experimental groups and confirmed that the BGF-treated mice showed a significant reduction in the MLI compared to the PBS/elastase-treated mice (Fig. 2D). These data suggest that BGF could improve the functional and structural alterations seen after elastase treatment in our mouse model.

### Hemodynamic amelioration of COPD-PH by BGF

**Fig. 3:**
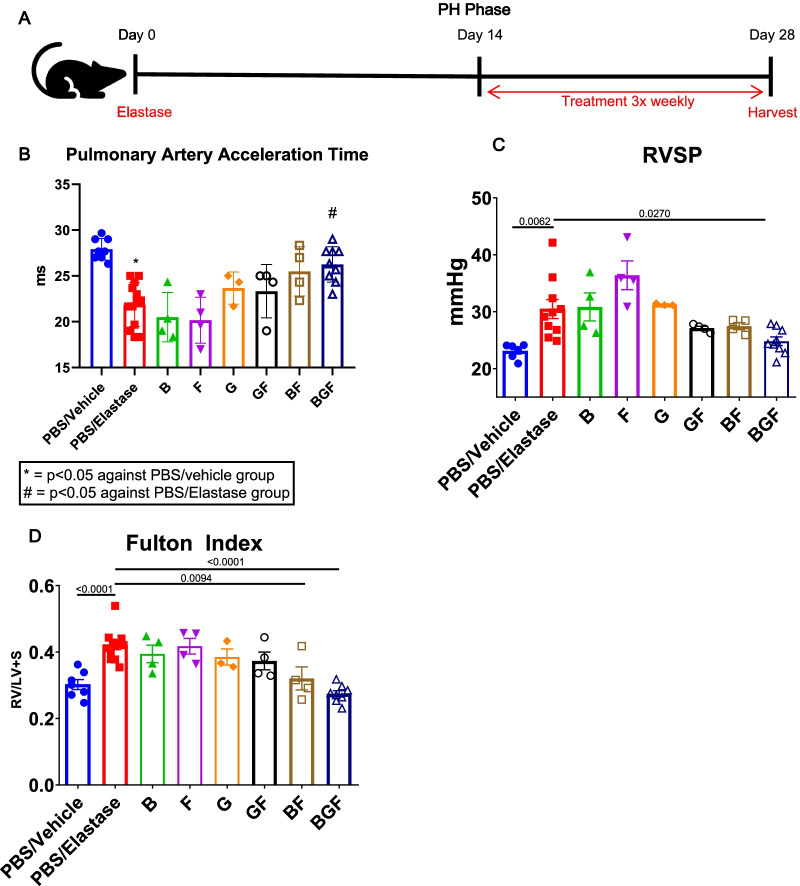
Prevention of pulmonary hypertension development in the BGF-treated groups. **A** Schematic diagram of the experimental design. BALB/c mice were treated with drug combinations or control vehicle on Day 14 after elastase treatment, hemodynamically analyzed, and sacrificed on Day 28. Lung and heart tissues were collected for further analysis. **B** Echocardiographic pulmonary artery acceleration time (PAAT) measurement results on Day 25. n = 3–13 mice per group. **C** Right ventricular systolic pressure (RVSP) measurement results via right heart catheterization just prior to termination on Day 28. n = 3–10 mice per group. **D** Right ventricular hypertrophy index (Fulton Index) measurement. n = 3–13 mice per group. The data are presented as the mean ± S.E.M. (standard error of mean). One-way ANOVA with Tukey’s post-hoc test was used for Figs. 3B-D. *, p < 0.05 compared with the PBS/Vehicle group, #, p < 0.05 compared with the PBS/Elastase group for (**B**)

To test our hypothesis about whether BGF treatment could positively affect PH development in COPD, we performed experiments in the pulmonary hypertension phase; to this end, we expanded on the previous elastase-induced emphysema mouse model and extended the duration of the post elastase instillation period to 28 days to allow the emphysematous mice to successfully develop PH. Similar to the treatments of the first two phases, we also treated the mice with the drug combinations or vehicle control three times weekly, this time starting on Day 14 (Fig. 3A).

Prior to termination at Day 28, noninvasive hemodynamic measurements of the mice using echocardiography were performed, and the results revealed a significant decrease in the pulmonary artery acceleration time (PAAT) after elastase treatment, while BGF treatment successfully prevented the decrease in the PAAT, achieving a time almost similar to that after PBS/vehicle treatment (Fig. 3B). At Day 28, invasive hemodynamic analysis of the mice just prior to termination and harvest using right heart catheterization to measure right ventricular systolic pressure (RVSP) revealed an increase in the RVSP in the PBS/elastase mice. The single drug-treated experimental mice failed to show any effect on the hemodynamics, while double drug-treated mice showed a reduction in pressure. Furthermore, BGF treatment successfully prevented the increase in the RVSP after elastase treatment (Fig. 3C). Consistent with the hemodynamic results, right ventricular hypertrophy measurement using the Fulton index (right ventricle/left ventricle + septum weight) showed that BGF treatment could prevent the development of right ventricular hypertrophy 28 days after elastase treatment (Fig. 3D). Additionally, BF-treated mice also showed a significant reduction in the Fulton index compared to the PBS/Elastase-treated mice (Fig. 3D). Taken together, these data showed that hemodynamic prevention of PH could be achieved after BGF treatment in our model.

### Prevention of pulmonary vascular remodeling after BGF treatment

A major process that contributes to PH development is hypoxia-driven pulmonary vascular remodeling, in which neomuscularization of the precapillary pulmonary arteries precedes the increase in pressure. Thus, we wanted to observe whether BGF could affect the remodeling of the lung vasculature that contributes to PH development. To this end, we analyzed the histological lung sections of the mice in the PH phase to determine whether there were any changes in the vascular structure.

**Fig. 4:**
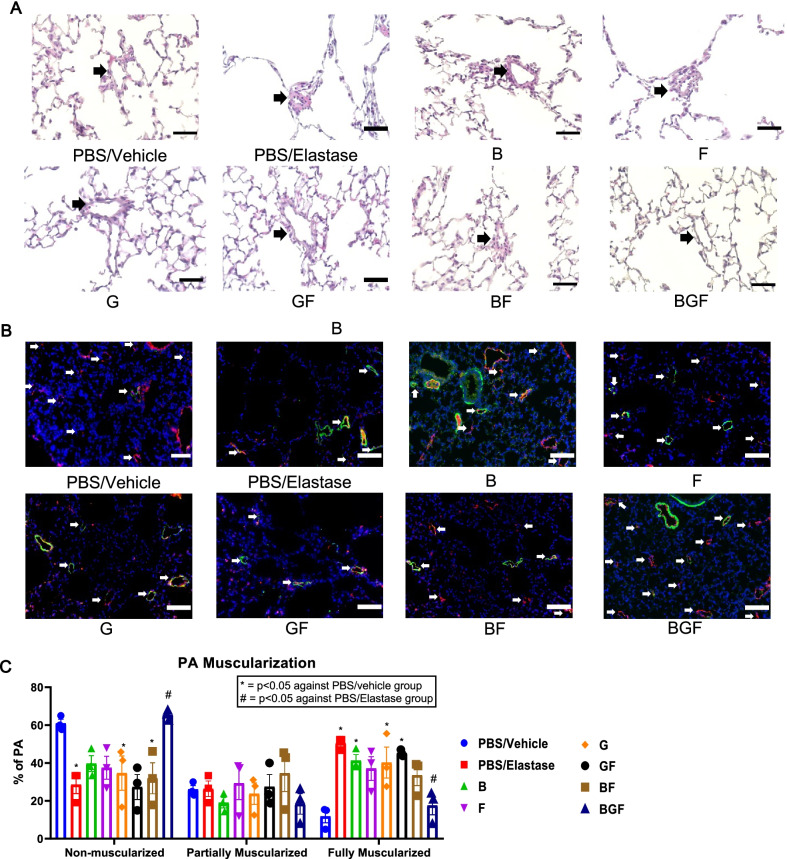
Improvement of pulmonary vascular remodeling in the PH phase in the BGF-treated groups. **A**, **B** Representative images of (**A**) hematoxylin–eosin staining (vessel indicated by black arrow) and **B** immunostaining of α-smooth muscle actin/α-SMA (green; stains smooth muscle cells) and von Willebrand factor/vWF (red; stains endothelial cells) in histological lung sections (vessel indicated by white arrows) from each experimental group. Scale bar: 50 μm for hematoxylin–eosin and 100 μm for immunostaining. **C** Quantification of distal pulmonary artery muscularization as determined by immunofluorescence staining of histological sections as described in the Methods section. n = 3 per group. The data are presented as the mean ± S.E.M. (standard error of mean). Two-way ANOVA with Tukey’s post-hoc test was used to analyze differences between each group, as shown in (**C**). *, p < 0.05 compared with the PBS/Vehicle group, #, p < 0.05 compared with PBS/Elastase in the respective nonmuscularized, partially muscularized and fully muscularized groups

While basic hematoxylin–eosin staining revealed a thickened precapillary vessel (< 50 μm in diameter) after elastase treatment that was not observed in the BGF treatment group (Fig. 4A), we sought to further confirm whether this was indeed due to muscularization of the precapillary arteries. Thus, we performed double immunostaining with anti-α-smooth muscle actin (α-SMA) antibodies to stain smooth muscle cells and anti-von Willebrand factor (vWF) antibodies to stain endothelial cells in the lung sections to visualize differences in muscularization. Here, we confirmed that muscularization of the precapillary arteries indeed occurred in the PBS/elastase group, and it was ameliorated after the drug treatments, especially after BGF treatment (Fig. 4B). When quantified, the results further showed that among the treatments, only the BGF treatment could significantly reduce the percentage of muscularized arteries compared with the PBS/elastase treatments (Fig. 4C). Thus, we believed that BGF treatment could ameliorate the pulmonary vascular remodeling observed in the PH phase after elastase treatment.

### BGF exerts its effect by inhibiting the major inflammatory signaling molecule NFκB in lung epithelial cells

To elucidate the molecular mechanism that could explain the in vivo effect of BGF treatment, we sought to identify pathways and molecules that could act as central factors to modulate the various processes that occur in COPD under both acute inflammatory and chronic emphysematous conditions. NFκB-mediated inflammation has been reported to be strongly involved in the pathogenesis of COPD while also having the ability to affect other COPD-related pathways. Thus, we aimed to prove that BGF could indeed affect this central inflammatory pathway.

**Fig. 5:**
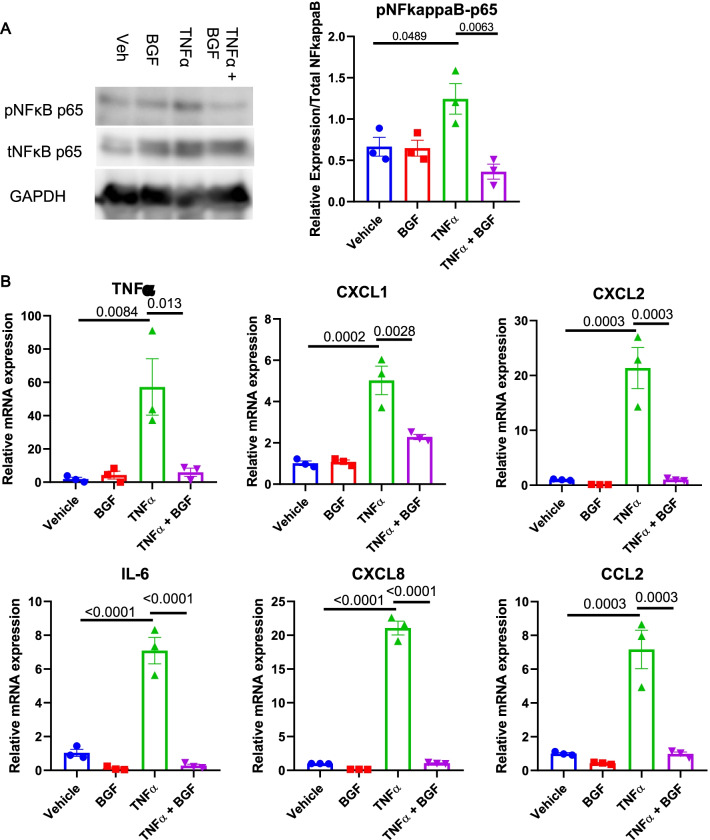
BGF treatment decreases NFκB phosphorylation and subsequent proinflammatory cytokine expression in lung epithelial cells. A549 cells were cultured until they reached ~ 90% confluence and treated with TNFα (10 ng/mL) or vehicle and with BGF or vehicle control for 3 h. Then, the cells were harvested and further analyzed. **A** Representative immunoblotting of phospho-NFκB p65, total NFκB p65 and GAPDH and the relative quantification (n = 3 biologically independent replicates) in A549 cells from all the experimental groups. Blotting images were cropped between 75 and 50 kDa for phospho-NFκB p65 and total NFκB p65, and between 37 and 25 kDa for GAPDH (**B**) Relative mRNA expression levels of TNFα, CXCL1, CXCL2, IL-6, CXCL8 and CCL2 in A549 cells from all the experimental groups. The data are presented as the mean ± S.E.M. (standard error of mean). One-way ANOVA with Tukey’s post-hoc test was used for all figures

First, we treated lung epithelial A549 cells with TNFα (10 ng/mL) or vehicle, and simultaneously added BGF or vehicle control for 3 h. Immunoblotting of NFκB p65 showed a marked increase in the levels of phosphorylated NFκB p65 after TNFα treatment, while the addition of BGF successfully abolished this phosphorylation (Fig. 5A).

A consequence of NFκB pathway activation is the increased expression of proinflammatory cytokines, and similar to the TNFα treatment experiment, we also analyzed whether there were differences in the production of cytokines after BGF treatment. Consistent with the immunoblotting results, TNFα induced increases in the mRNA levels of cytokines (levels of TNFα, CXCL1, CXCL-2, IL-6, CXCL-8 and CCL2 shown here), and these effects were successfully abolished by BGF treatment (Fig. 5B). We concluded that BGF treatment could effectively limit proinflammatory reactions in lung epithelial cells through NFκB pathway inhibition.

### Direct effects of BGF on NFκB inhibition in lung endothelial cells

Because prolonged hypoxia and inflammation could also adversely affect endothelial cells and promote vascular remodeling and we previously found that BGF could ameliorate vascular remodeling and subsequent PH development in vivo, we postulated that in addition to its benefit in epithelial cells, BGF treatment could also directly affect lung endothelial cells through the NFκB pathway.

**Fig. 6:**
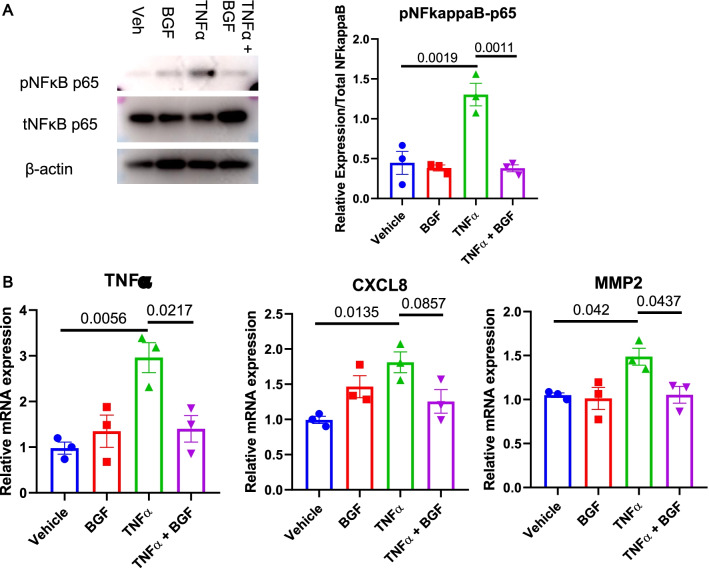
Direct anti-inflammatory effect of BGF treatment on lung endothelial cells via NFκB inhibition. Mouse lung endothelial cells (MLECs) were isolated via magnetic cell sorting, cultured until they reached ~ 90% confluence and treated with TNFα (10 ng/mL)/vehicle and with BGF/vehicle for 24 h. Then, the cells were harvested and further analyzed. **A** Representative immunoblotting of phospho-NFκB p65, total NFκB p65 and β-actin and the relative quantification (n = 3 biologically independent replicates) in MLECs from all the experimental groups. Blotting images were cropped between 75 and 50 kDa for phospho-NFκB p65 and total NFκB p65, and between 50 and 37 kDa for β-actin (**B**) Relative mRNA expression levels of TNFα, CXCL-8 and MMP2 in MLECs from all the experimental groups. The data are presented as the mean ± S.E.M. (standard error of mean). One-way ANOVA with Tukey’s post-hoc test was used for all figures

We first analyzed whether the phosphorylation of NFκB p65 could also be abolished by BGF treatment in lung endothelial cells by treating isolated mouse lung endothelial cells (MLECs) with similar concentrations of TNFα (10 ng/mL) or vehicle and with BGF or vehicle for 24 h. We also observed decreased levels of phosphorylated NFκB p65 in MLECs after BGF treatment, even in the presence of TNFα (Fig. 6A). The increase in the mRNA expression of cytokines after TNFα treatment could also be reversed by BGF treatment in MLECs, consistent with the findings in A549 cells (Fig. 6B). To observe the effects of BGF on other potential signaling pathways, we performed immunostaining for eNOS, VEGF-A, and Ki67 on lung histological sections from all experimental groups on the PH phase. While the increase in Ki67-positive vasculature could be ameliorated by BGF treatment, neither elastase-induced increase in tissue eNOS or VEGF-A expression were significantly changed after BGF treatment (Additional file 2: Fig. S2). We finally concluded that BGF could have a direct effect on MLECs through a similar mechanism of NFκB inactivation.

### Budesonide-containing treatments exert more potent anti-inflammatory effects in lung epithelial cells

**Fig. 7:**
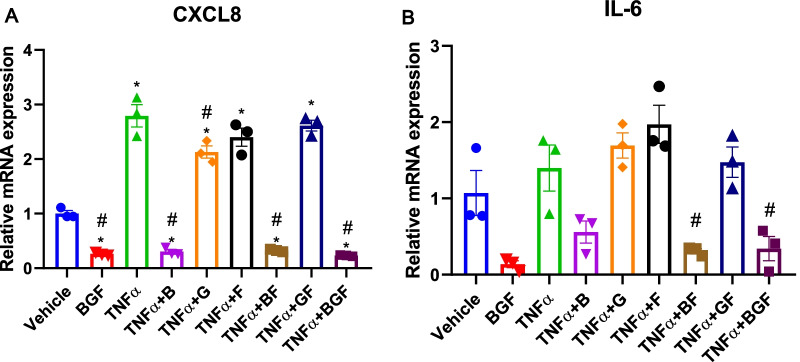
Budesonide-containing treatments preferentially exerted anti-inflammatory effects on lung epithelial cells. A549 cells were cultured until they reached ~ 90% confluence and treated with TNFα (10 ng/mL) or vehicle and with vehicle or drug combinations (B, G, F, BF, GF, and BGF) for 3 h. Then, the cells were harvested and further analyzed. **A**, **B** Relative mRNA expression levels of IL-6 (A) and CXCL-8 (**B**) in A549 cells from all the experimental treatment groups (n = 3 biologically independent replicates). The data are presented as the mean ± S.E.M. (standard error of mean). One-way ANOVA with Tukey’s post-hoc test was used for all figures. *, p < 0.05 compared with the PBS/Vehicle group, #, p < 0.05 compared with the PBS/TNFα group for all figures

To determine which drug or drug combinations have the greatest influence in mediating the anti-inflammatory effect of BGF, we again performed a similar 3-h TNFα treatment experiment in A549 cells. In this experiment, we expanded the treatment groups to include the B, G, F, BF and GF groups, similar to the in vivo experiment. Quantitative real-time PCR analysis revealed that of the two vital COPD-related cytokines that we analyzed (CXCL8 and IL-6), only the budesonide-containing treatments, namely, the BF and BGF treatments, could notably reverse the TNFα-induced increases in cytokine expression level (Fig. 7A, B). We thus concluded that among our experimental treatments, budesonide-containing treatments exert the strongest anti-inflammatory effects (Additional file 3).

## Discussion

Although PH development and eventual right heart failure are one of the major causes of morbidity and mortality in COPD patients, current therapeutic options that can specifically and efficiently address COPD-PH conditions are rare [31]. Here, we present a potential novel strategy based on the inhalation of triple therapies that could help treat this condition while providing molecular evidence about how such treatments might act on lung cells.

It is interesting to see not only that BGF treatment is confirmed to be effective in both of the COPD phases (acute inflammatory and emphysematous phases) but also that it can be effective in preventing further development of PH. Although other drug combinations either failed or did not consistently improve the COPD and PH conditions, in our study, BGF treatment exerted a consistent effect in treating those conditions.

Inflammation, both acute and chronic inflammation, is a common mechanism in COPD and subsequent PH development [32, 33]. As such, we believe that it could also serve as a link between the pathogenesis of COPD and PH. BGF treatment is effective in preventing inflammation in epithelial cells, and other studies of individual drugs have shown similar results. BGF treatment also exerts a direct effect in ameliorating proinflammatory responses in ECs. This suggests a possible common mechanism underlying the effect of BGF in treating this pathway. We also found that multiple proinflammatory cytokines are affected by BGF treatment, and this evidence possibly suggests that a central regulator of cellular inflammatory reactions is involved in mediating the molecular activity of BGF. Indeed, we found that BGF exerted its effects through a similar mechanism involving the NFκB pathway in both A549 cells and MLECs. The transcription factor NFκB is already known to trigger cellular inflammatory reactions after activation by various inflammatory stimuli, and its nuclear translocation, which is mediated by phosphorylation of p50 and p65 in addition to IκBα that prevents the translocation, can trigger the mRNA expression of a host of proinflammatory cytokines [34, 35]. Previous reports have also linked the NFκB pathway to PH [20]. Our study showed that BGF can directly ameliorate this pathway in both cell lines, suggesting that this compound may have a common molecular mechanism that can ultimately exert similar effects regardless of cell type. We did not observe any changes, however, to other PH and/or COPD-related pathways that could be related to the effect of BGF treatment, such as NO or VEGF pathway, while the observed changes in the vascular proliferation capability could also be attributed to the alteration in NFκB pathway [36–40].While individual drugs are known to affect NFκB activation and subsequent cytokine production, further molecular studies are warranted to elucidate this mechanism [41].

One aim of our study was to analyze whether there is a preferable combination that can differentially affect lung cells in different phases. While BGF seemed to exert a synergistic effect in preventing proinflammatory reactions in all experimental phases that was not exerted by other drugs, interestingly, dual combination therapy, such as BF or GF, did not exert a protective effect consistent with that of BGF in vivo, although GF treatment significantly improved the acute inflammatory reaction, and BF improved right ventricular hypertrophy development under prolonged emphysematous conditions. However, the synergistic effect that dual or triple combination therapy had compared to single drug treatment groups are apparent, although it did not reach statistical significance in all phases experiments. This could be explained by the interactions and synergism between steroids and β agonists in exerting anti-inflammatory effects, and between formoterol and glycopyrronium in affecting different pathways of anti-inflammatory and bronchodilating effects [42, 43]. Additionally, it is interesting to note is that although the dual drug combinations showed tendencies of improving emphysematous and PH conditions, the effects were relatively milder and less consistent than those of the BGF combination treatment. Moreover, the results from these three phases suggest that different drugs can positively affect COPD and COPD-PH conditions in different manners. A prime example of this is how GF could possibly ameliorate inflammatory conditions but not show any tendency toward improving the histological lung structure.

Further elaborating on the differing functions of the drugs and how they could work alone or together, by comparing combinations in vitro, we found that combinations that included budesonide had the strongest effects on the expression of proinflammatory cytokines, as expected for a potent anti-inflammatory agent [44]. We decidedly selected two specific cytokines, IL-6 and CXCL8, because both cytokines are important in both COPD and PH [45–48]. Notably, there was a decreasing trend in their expression with the addition of formoterol and a further reduction with the addition of glycopyrronium. One possible explanation for this synergistic effect, at least in the BF group, is the aforementioned mutually beneficial interactions between steroids and β agonist agents; β agonists could possibly improve steroid-mediated cytokine repression, while steroids help upregulate β receptor mRNA expression levels [43]. However, considering that studies on the mechanism underlying the combined use of both formoterol and glycopyrronium with budesonide are rare, we believe that the interaction of these drugs and the seemingly positive synergistic effect of the BGF combination warrants further studies in the future [49].

Increasing evidence has shown the benefit of BGF inhalation treatment in COPD patients, culminating in the recent approval of this drug combination as a therapeutic option for COPD treatment [11]. In our study, we not only confirmed the efficacy of BGF in COPD but also showed the additional benefit of BGF for COPD-PH conditions. The usage of the drug classes in our study, especially LAMA and LABA drugs, to specifically treat type 3 PH has not been widely studied in either basic or clinical settings. Corticosteroids, on the other hand, have been reported to be effective in treating PH, both in basic and clinical studies [50]. Specifically, dexamethasone has been reported to be effective in reversing monocrotaline-induced PH, while other reports showed the efficiency of prednisolone in PAH patients [50–52]. Nevertheless, there is a notable lack of clinical data related to the usage of corticosteroids, especially budesonide, in PH. As such, we hope that the results of our study will contribute to filling the knowledge gap on this specific topic.

## Conclusion

In conclusion, the results of our study contributed to the increasing evidence that suggests the benefits of BGF treatment in COPD while also suggesting that BGF treatment could also be effective in preventing the occurrence of COPD-related PH. Further studies regarding the clinical efficacy of BGF in COPD-PH and further research on the detailed molecular mechanism underlying the usage of COPD drug classes to treat COPD-PH, specifically the mechanism associated with this specific BGF drug combination, could potentially provide a novel way to treat COPD-PH patients in the future.

## Supplementary Information

**Additional file 1: Table S1.** List of primers used for qPCR analysis.

**Additional file 2: Figure S1.** Lymphocyte counts in the BAL fluid of mice from all experimental groups 1 day post-elastase treatment. **Figure S2.** Immunostaining VEGF-A (A), eNOS (B), and Ki 67 (C) in the lung vasculature of mouse lung of all experimental groups at Pulmonary Hypertension phase. Scale bar:50 uM. Black arrows indicate vessel.

**Additional file 3.** Uncut blotting images for Budesonide/Glycopyrronium /Formoterol Fumarate Triple Therapy Prevents Pulmonary Hypertension in a COPD Mouse Model via NFκB Inactivation.

## Data Availability

The datasets used and/or analyzed during the current study are available from the corresponding author on reasonable request.

## Abbreviations

COPD: Chronic obstructive pulmonary hypertension
PH: Pulmonary hypertension
B: Budesonide
G: Glycopyrronium
F: Formoterol fumarate
BGF: Budesonide + glycopyrronium + formoterol fumarate
BALF: Bronchoalveolar lavage fluid
PBS: Phosphate-buffered saline
NFκB: Nuclear factor kappa-light-chain-enchancer of activated B cells
PAH: Pulmonary arterial hypertension
ICS: Inhaled corticosteroids
LAMA: Long-acting muscarinic antagonist
LABA: Long-acting beta agonist
NO: Nitric oxide
VEGF: Vascular endothelial growth factor
EC: Endothelial cell
TNFα: Tumor necrosis factor alpha
IL-6: Interleukin 6
IL-1β: Interleukin 1 beta
CCL1: C–C motif chemokine ligand 1
CCL2: C–C motif chemokine ligand 2
CCL8: C–C motif chemokine ligand 8
BAL: Bronchoalveolar lavage
MLI: Mean linear intercept
PAAT: Pulmonary artery acceleration time
RVSP: Right ventricular systolic pressure
α-SMA: Alpha smooth muscle actin
VWF: Von Willebrand factor
MLEC: Mouse lung endothelial cells
CXCL-1: C-X-C motif chemokine ligand 1
CXCL-2: C-X-C motif chemokine ligand 2
CXCL-8: C-X-C motif chemokine ligand 8
