# Supplementary material for: Budesonide/glycopyrronium/formoterol fumarate triple therapy prevents pulmonary hypertension in a COPD mouse model via NFκB inactivation
Source: Respir Res. 2022 Jun 27;23:173. doi: 10.1186/s12931-022-02081-y (PMC9238100; doi:10.1186/s12931-022-02081-y)
Supplement: Supplementary file 1 — Additional file 1: Table S1. List of primers used for qPCR analysis. [file 12931_2022_2081_MOESM1_ESM.docx]

**Additional File 1**

**Additional Table 1.** List of Primers used for qPCR analysis

| **Gene** | |
| --- | --- |
| *Human* | |
| GAPDH | Forward: 5'-gcaccgtcaaggctgagaac-3' |
|  | Reverse: 5'-atggtggtgaagacgccagt-3' |
| CXCL1 | Forward: 5'-aaccgaagtcatagccacac-3' |
|  | Reverse: 5'-gttggatttgtcactgttcagc-3' |
| CXCL2 | Forward: 5'-cccaagttagttcaatcctg-3' |
|  | Reverse: 5'-ttcctcagcctctatcacag-3' |
| TNFα | Forward: 5'-agagggaagagttccccagggac-3' |
|  | Reverse: 5'-tgagtcggtcacccttctccag-3' |
| CXCL8 | Forward: 5'-gcataaagacatactccaaacc-3' |
|  | Reverse: 5'-acttctccacaaccctctg-3' |
| CCL2 | Forward: 5'-cagccagatgcaatcaatgcc-3' |
|  | Reverse: 5'-tggaatcctgaacccacttct-3' |
| IL-6 | Forward: 5'-ggtacatcctcgacggcatct-3' |
|  | Reverse: 5'-gtgcctctttgctgctttcac-3' |
| *Mouse* | |
| β-Actin | Forward: 5'-atgaagatcaagatcattgctcctc-3' |
|  | Reverse: 5'-acatctgctggaaggtggacag-3' |
| CXCL2 | Forward: 5'-gcccagacagaagtcatagcc-3' |
|  | Reverse: 5'-ctcctcctttccaggtcagtta-3' |
| IL-1β | Forward: 5'-tgggaaacaacagtggtcagg-3' |
|  | Reverse: 5'-ccatcagaggcaaggaggaa-3' |
| TNFα | Forward: 5'-actgaacttcggggtgatcggtcc-3' |
|  | Reverse: 5'-gtgggtgaggagcacgtagtcg-3' |
| CXCL8 | Forward: 5'-tggctgggattcacctcaa-3' |
|  | Reverse: 5'-gagtgtggctatgacttcggttt-3' |
| MMP2 | Forward: 5'-ggtgaaggtcggtgtgaacggattt-3' |
|  | Reverse: 5'-aatgccaaagttgtcatggatgacc-3' |
| IL-6 | Forward: 5'-gatctcgaatgaagacccctggc-3' |
|  | Reverse: 5'-aaggactctggctttgtctttct-3' |
