# Supplementary material for: Budesonide/glycopyrronium/formoterol fumarate triple therapy prevents pulmonary hypertension in a COPD mouse model via NFκB inactivation
Source: Respir Res. 2022 Jun 27;23:173. doi: 10.1186/s12931-022-02081-y (PMC9238100; doi:10.1186/s12931-022-02081-y)
Supplement: Supplementary file 2 — Additional file 2: Figure S1. Lymphocyte counts in the BAL fluid of mice from all experimental groups 1 day post-elastase treatment. Figure S2. Immunostaining VEGF-A (A), eNOS (B), and Ki 67 (C) in the lung vasculature of mouse lung of all experimental groups at Pulmonary Hypertension phase. Scale bar:50 uM. Black arrows indicate vessel. [file 12931_2022_2081_MOESM2_ESM.pdf]

Additional File 2
Additional Figures for "Budesonide/Glycopyrronium/Formoterol Fumarate Triple Therapy Prevents Pulmonary Hypertension in a COPD Mouse Model via NFκB Inactivation"

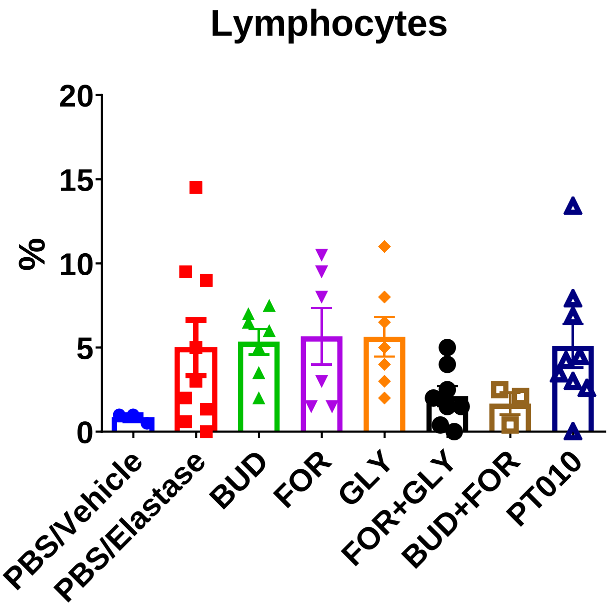

Additional Figure 1. Lymphocyte counts in the BAL fluid of mice from all experimental groups 1 day post-elastase treatment.

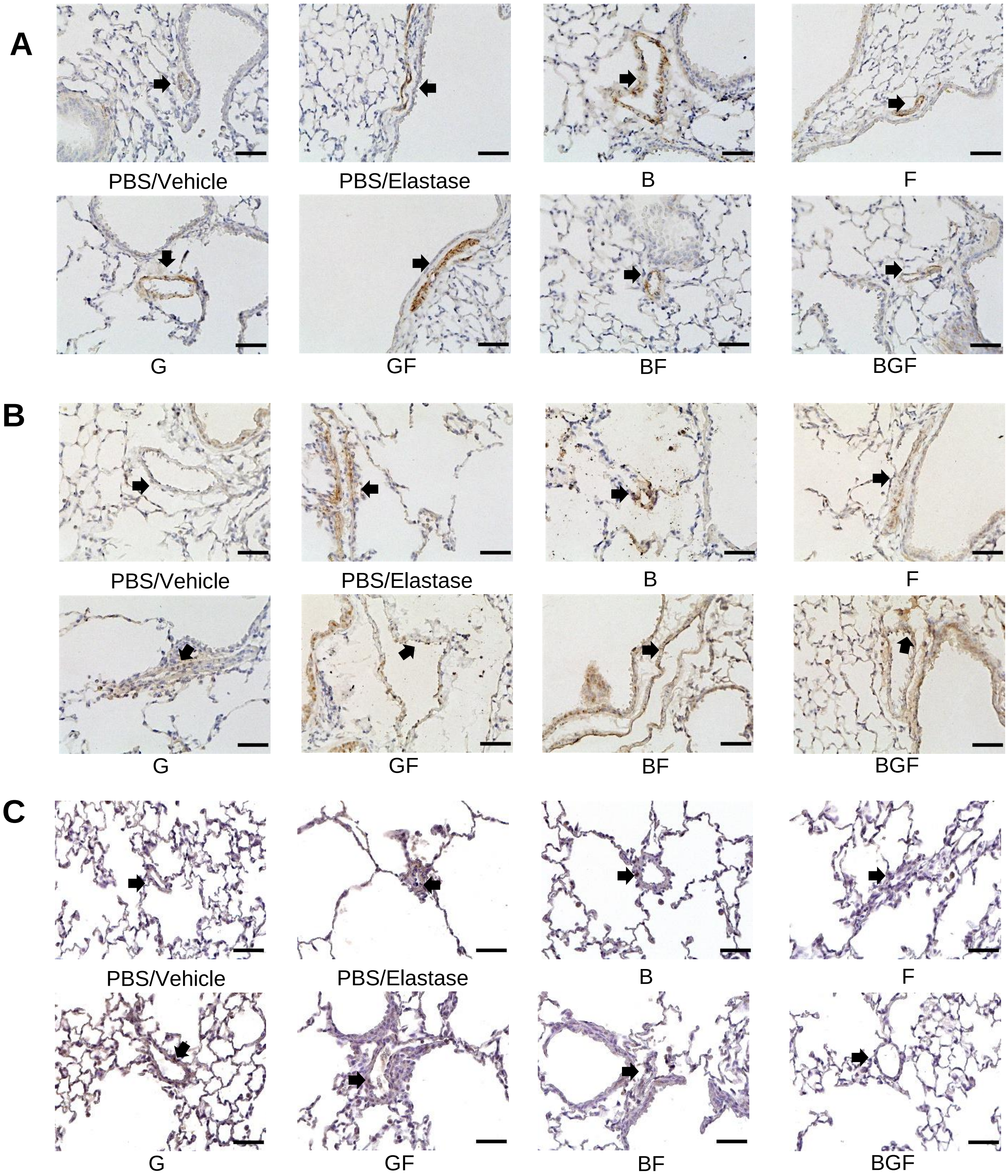

Additional Figure 2. Immunostaining of VEGF-A (A), eNOS (B), and Ki67 (C) in the lung vasculature of mouse lung of all experimental groups at Pulmonary Hypertension phase. Scale bar: 50 uM. Black arrows indicate vessel.
