# Supplementary material for: Budesonide/glycopyrronium/formoterol fumarate triple therapy prevents pulmonary hypertension in a COPD mouse model via NFκB inactivation
Source: Respir Res. 2022 Jun 27;23:173. doi: 10.1186/s12931-022-02081-y (PMC9238100; doi:10.1186/s12931-022-02081-y)

Additional File 3
Uncut blotting images for “Budesonide/Glycopyrronium/Formoterol Fumarate Triple Therapy Prevents Pulmonary Hypertension in a COPD Mouse Model via NFκB Inactivation”

Figure 5A

Phospho-NFκB

250 kDa
150 kDa
100 kDa
75 kDa
50 kDa

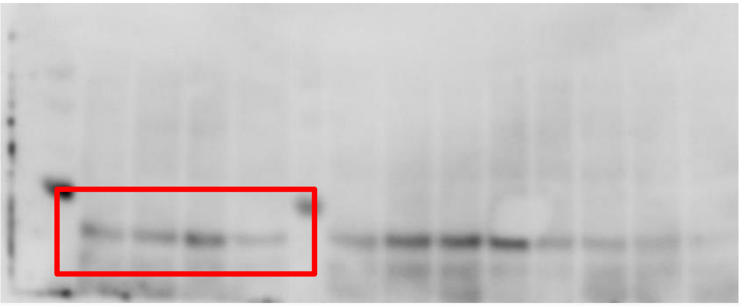

Total-NFκB

250 kDa
150 kDa
100 kDa
75 kDa
50 kDa

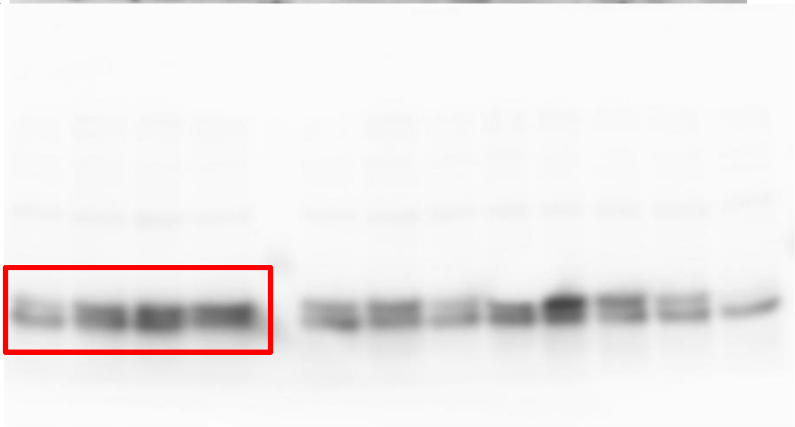

GAPDH

50 kDa
37 kDa
25 kDa

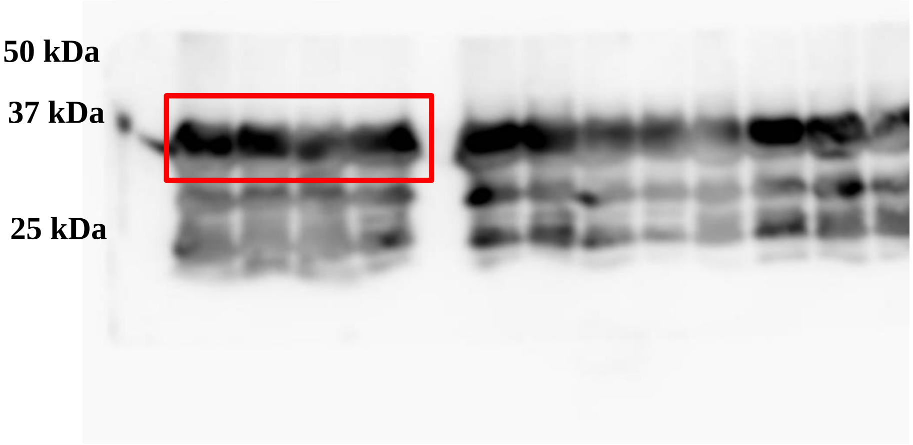

Figure 6A

Phospho-NFκB

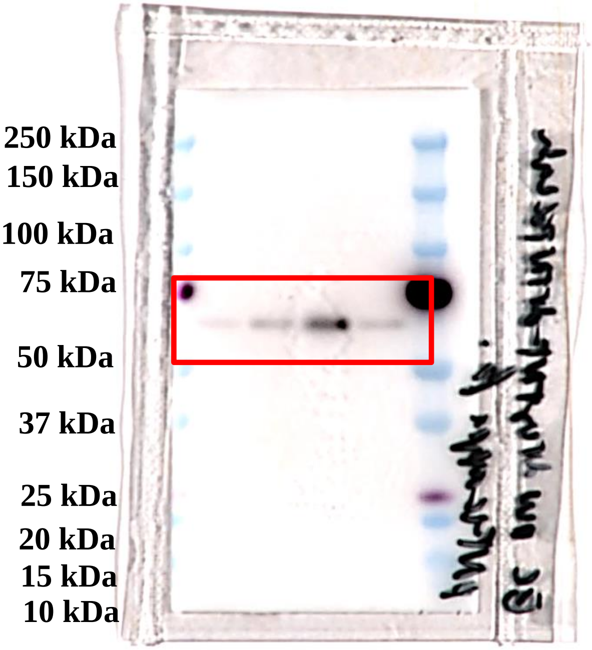

Total-NFκB

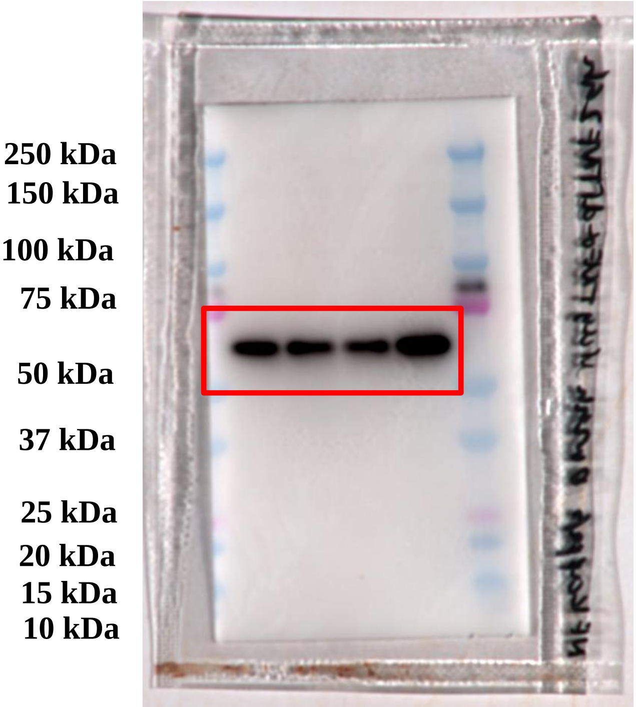

β-actin

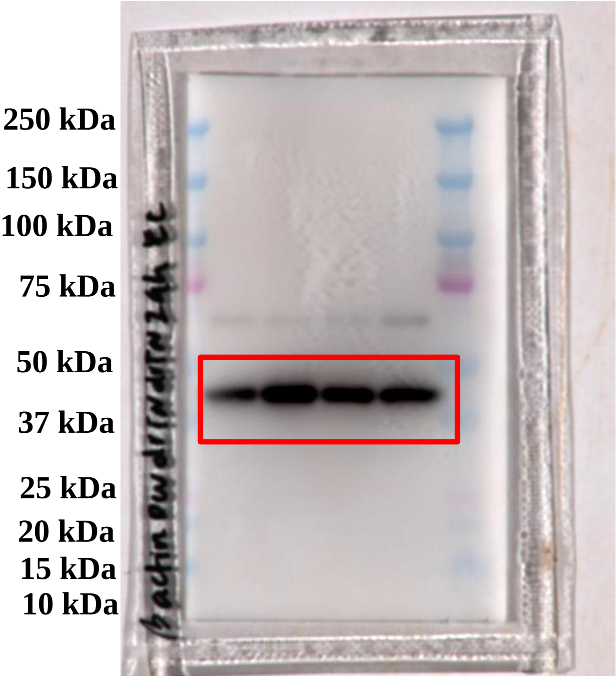

Supplement: Supplementary file 3 — Additional file 3. Uncut blotting images for Budesonide/Glycopyrronium /Formoterol Fumarate Triple Therapy Prevents Pulmonary Hypertension in a COPD Mouse Model via NFκB Inactivation. [file 12931_2022_2081_MOESM3_ESM.pdf]
